# Isaacs’ syndrome as the initial presentation of malignant thymoma and associated with double-positive voltage-gated potassium channel complex antibodies, a case report

**DOI:** 10.1186/s12883-022-02584-7

**Published:** 2022-03-04

**Authors:** Kuan-Ching Li, Ming-Feng Liao, Yih-Ru Wu, Rong-Kuo Lyu

**Affiliations:** Department of Neurology, Chang Gung Memorial Hospital Linkou Medical Center and Chang Gung University College of Medicine, No.5, Fusing St., Gueishan Township, Taoyuan County, Taiwan

**Keywords:** Isaacs’ syndrome, Thymoma, Leucine-rich glioma-inactivated 1 (LGI1) antibody, Contactin-associated protein 2 (CASPR2) antibody, Case report

## Abstract

**Background:**

Isaacs’ syndrome is a peripheral nerve hyperexcitability (PNH) syndrome due to peripheral motor nerve instability. Acquired Isaacs’ syndrome is recognized as a paraneoplastic autoimmune disease with possible pathogenic voltage-gated potassium channel (VGKC) complex antibodies. However, the longitudinal correlation between clinical symptoms, VGKC antibodies level, and drug response is still unclear.

**Case presentation:**

A 45-year-old man had progressive four limbs soreness, muscle twitching, cramps, and pain 4 months before admission. Electromyography (EMG) studies showed myokymic discharges, neuromyotonia, and an incremental response in the high-rate (50 Hz) repetitive nerve stimulation (RNS) test. Isaacs’ syndrome was diagnosed based on clinical presentations and EMG reports. Serum studies showed positive VGKC complex antibodies, including leucine-rich glioma-inactivated 1 (LGI1) and contactin-associated protein-like 2 (CASPR2) antibodies. The acetylcholine receptor antibody was negative. Whole-body computed tomography (CT) and positron emission tomography revealed a mediastinal tumor with the great vessels encasement, right pleura, and diaphragm seeding. Biopsy confirmed a World Health Organization type B2 thymoma, with Masaoka stage IVa. His symptoms gradually improved and both LGI1 and CASPR2 antibodies titer became undetectable after concurrent chemoradiotherapy (CCRT) and high dose steroid treatment. However, his Isaacs’ syndrome recurred after the steroid was reduced 5 months later. Follow-up chest CT showed probable thymoma progression. LGI1 antibody turned positive again while CASPR2 antibody remained undetectable.

**Conclusions:**

Our patient demonstrates that Isaacs’ syndrome could be the initial and only neuromuscular manifestation of malignant thymoma. His Isaacs’ syndrome is correlated well with the LGI1 antibody level. With an unresectable thymoma, long-term immunosuppressant therapy may be necessary for the management of Isaacs’ syndrome in addition to CCRT for thymoma.

## Background

Isaacs’ syndrome is a peripheral nerve hyperexcitability (PNH) syndrome that is characterized by continuous muscle twitching, myokymia, muscle hypertrophy, and dysautonomia, and sometimes associated with neuropathic pain and paresthesia. Electrodiagnostic tests had unique spontaneous motor activities including fasciculation, myokymic discharges, and neuromyotonic discharge [1]. Presently, an acquired Isaacs’ syndrome is recognized as a paraneoplastic autoimmune condition that correlates with voltage-gated potassium channels (VGKC) complex antibodies and underlying tumors, especially thymoma [2]. However, marked clinical overlaps are found between patients with different antibodies, including leucine-rich glioma-inactivated 1 (LGI1) and contactin-associated protein-like 2 (CASPR2) antibodies, the most common VGKC complex antibodies [3, 4]. The association between autoantibodies titer and treatment response is seldom documented. Besides while immunosuppressant therapy has shown a beneficial effect, whether long-term treatment is necessary for these patients remains undetermined.

Here we report a patient with malignant thymoma that initially presented with Isaacs’ syndrome, focusing on the relationship between clinical symptoms, serum autoantibodies titer, response to immunosuppressant therapy, and concurrent thymoma condition.

## Case presentation

A 45-years-old man who denied systemic disease presented progressive four limbs soreness 4 months before admission. He developed nuchal soreness at first then soreness radiated to bilateral shoulders and bilateral upper arms. His muscle twitching insidiously developed on the bilateral upper limbs, lips, and bilateral shoulders one month after symptoms onset. He also noticed numbness from the right distal fingers. Gradually, both his lower legs exhibited similar symptoms in the following months. Moreover, his muscle started to cramp, especially in bilateral thighs and calves, and his toes frequently encountered flexion cramps. His lower limbs became weak three days before admission and he had difficulties in walking for a long distance, standing for a long time, and climbing upstairs. He also had progressive four limbs twitching, cramps, fullness sensation, and pain that bothered his daily activity and interrupted his sleep. On neurological examination, he had proximal muscle weakness of four limbs with a Medical Research Council’s scale grade 4. He also had a positive Gowers’ sign. We noticed fasciculation and myokymia in his four limbs, especially in bilateral calf muscles. His cranial nerve function, sensation including thermal and proprioception, coordination, and deep tendon reflexes remained intact.

Routine blood analysis showed mild elevation of liver enzymes (Aspartate transaminase 47 U/L, normal ≦34 U/L; Alanine aminotransferase 105 U/L, normal ≦36 U/L), creatine kinase (302 U/L, normal range: 20–200 U/L), and lactate dehydrogenase (344 U/L, normal range: 98 ~ 192 U/L). Other serum examinations including tumor marker (cancer antigen 19–9, carcinoembryonic antigen, prostate-specific antigen, alpha-fetoprotein), thyroid function and adrenal function, antinuclear antibody, heavy metals (copper, zinc, lead, calcium), hepatitis B, and hepatitis C titer were in the normal ranges. His cerebrospinal fluid analysis revealed normal cellular count, protein, and sugar levels.

**Fig. 1:**
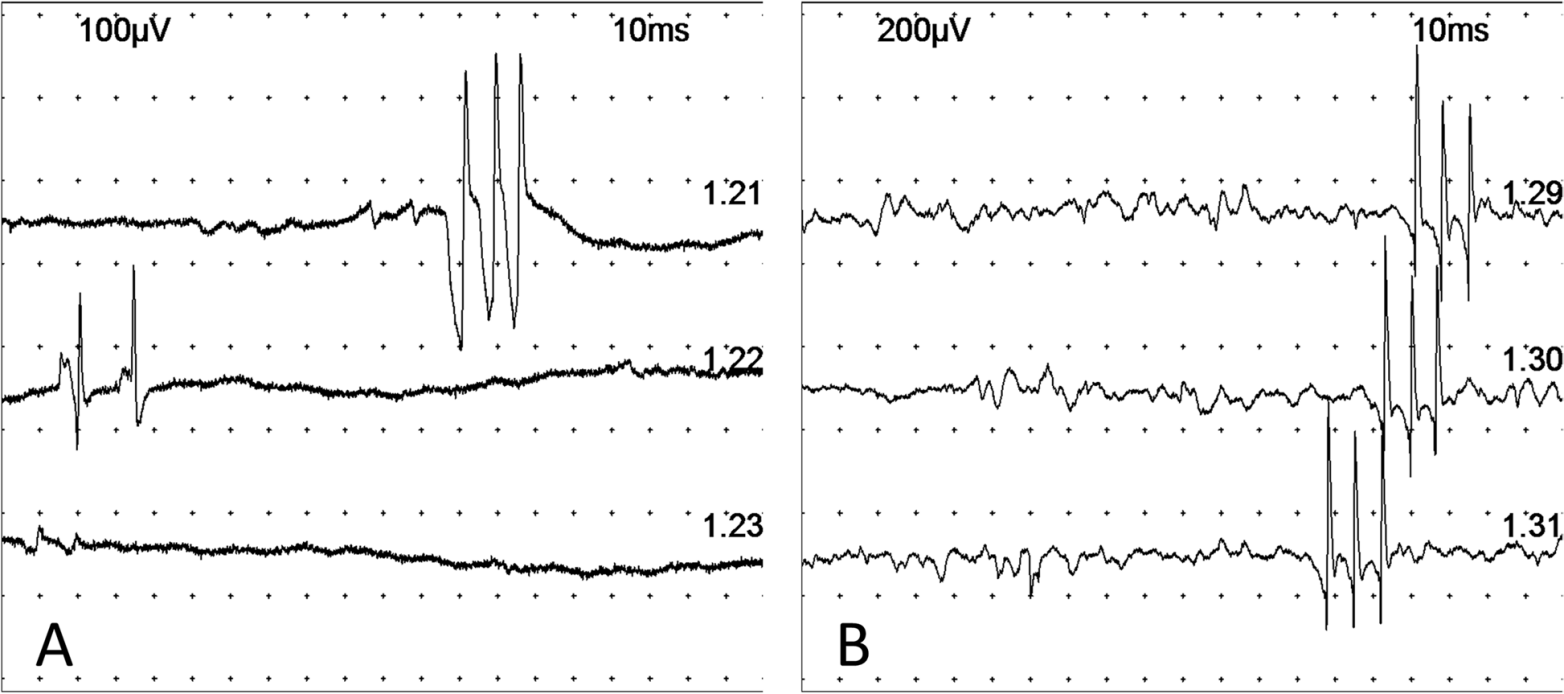
Resting Needle Electromyography (EMG). The resting needle EMG of this patient showed classical triplets motor unit action potential at the left gastrocnemius muscle (**A**) and left abductor digiti minimi muscle (**B**)

Electromyography (EMG) studies showed spontaneous motor unit discharge including fasciculation, neuromyotonic discharge, and myokymic discharge (triplet and doublet) at rest (Fig. 1). High-rate (50 Hz) repetitive nerve stimulation (RNS) test showed an incremental response (102%) at the left abductor digiti minimi muscle, which suggested presynaptic neuromuscular junction dysfunction. (Table 1).

**Table 1:** Summary of clinical symptoms, tumor progression, and antibodies titer

Date	2020/04	2020/07	2020/11
Clinical syndrome	Bilateral calf and thigh muscle cramping and twitching.	Bilateral calf and thigh muscle cramping improved.	Bilateral calf and thigh muscle twitching recurred.
LGI1^a^ antibody titer	1:10	Negative	1:10
CASPR2^b^ antibody titer	1:100	Negative	Negative
Chest computed tomography	Malignant thymoma (8.7 cm) with pleural and great vessel involvement.	Malignant thymoma with regression (4.5 cm).	Malignant thymoma with suspicion of post-radiation changes or progression (5.7 cm).
High-rate (50%) repetitive nerve stimulation test	Incremental response (102%)	Normal	Nil

^a^*LGI1* leucine-rich glioma-inactivated 1

^b^ *CASPR2* contactin-associated protein-like 2

**Fig. 2:**
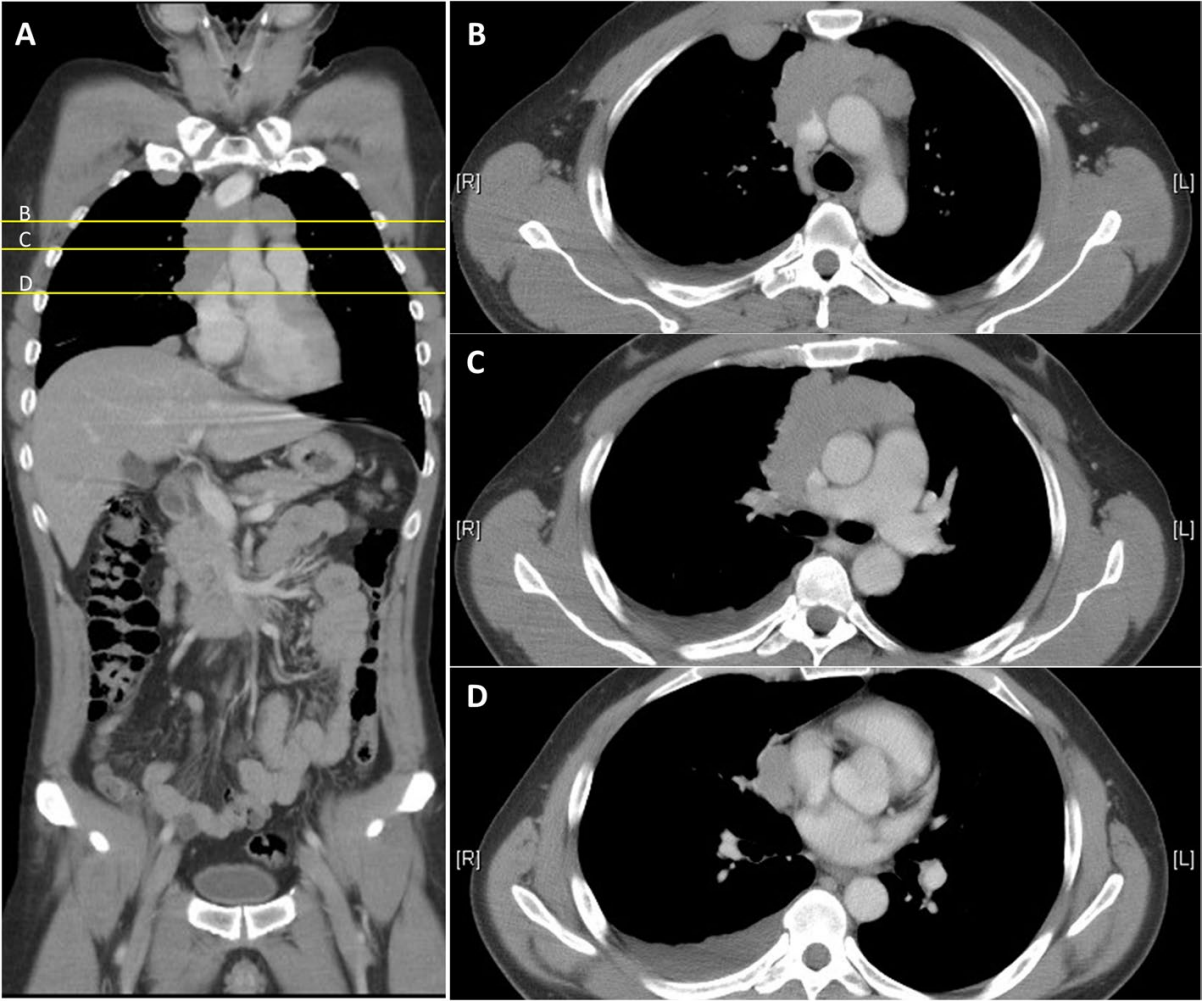
Whole-body computed tomography (CT) image. The coronal whole-body CT study of this patients (**A**) showed an enhancing mass lesion (8.7 × 3.9 cm) at the anterior mediastinum, which is suspected as a thymoma, with tumor attachment/invasion of ascending aorta, pulmonary artery, and partial encasement of superior vena cava (**B**, **C**). Nodular thickening of the right pleura (**B**) and right-side pericardium (**D**), which are suspected tumor invasions are also noted

**Fig. 3:**
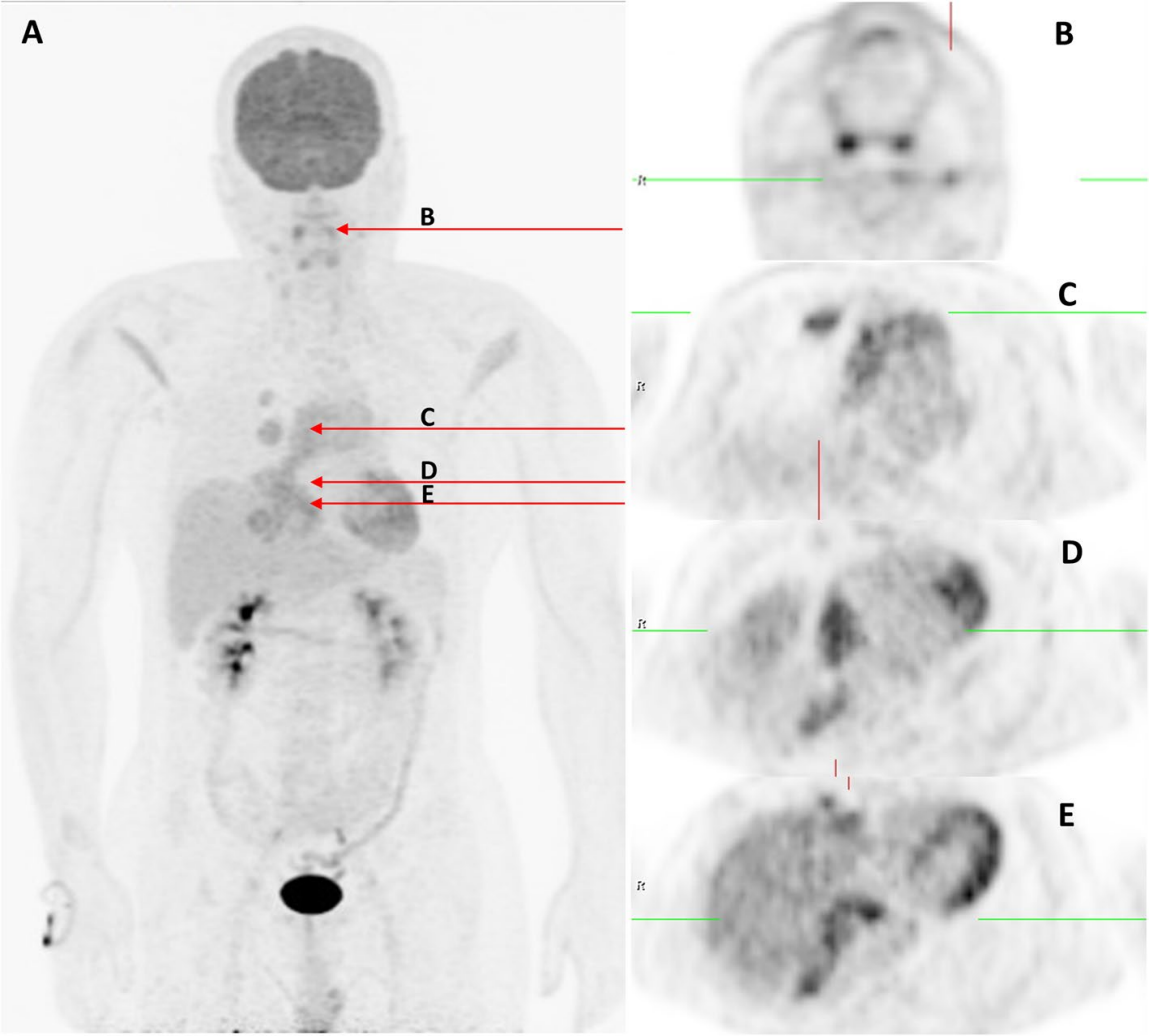
Positron emission tomography (PET) of the whole body. The PET of the whole body (**A**) showed the tumor with anterior mediastinum (**C**), right pleura (**C**), diaphragm invasions (**D**, **E**), and bilateral neck lymph node lesions (**B**), which are probable metastatic lymphadenopathy or reactive lymphadenopathy

Autoantibody to acetylcholine receptor was negative. Moreover, we cooperate with UniPharma company and used the cell-based indirect immunofluorescence assay (EUROIMMUN, Lubeck, Germany) to detect serum antibody, which showed positive LGI1 (1:10, results with a titer 1:< 10 were considered negative) and CASPR2 (1:100, results with a titer 1:< 100 were considered negative) antibodies. (Table 1) The whole-body computed tomography (CT) scan showed an enhancing mass lesion (8.7 × 3.9 cm) at anterior mediastinum, suspected malignant thymoma with great vessel attachment, and tumor seeding at right pleural and right-side pericardium (Fig. 2). Positron emission tomography scan showed the tumor involving the anterior mediastinum, right pleura, and diaphragm, and bilateral neck lymph node lesions suspected reactive or metastasis (Fig. 3). The pathologic report of CT-guided biopsy was consistent with thymoma (WHO type B2). The clinical stage showed Masaoka stage IVa, and American Joint Committee on Cancer (AJCC) stage IV (T4N2M1). The oncologist suggested concurrent chemoradiotherapy (CCRT) with chemotherapy (doxorubicin, cisplatin, vincristine, and cyclophosphamide [ADOC]) every 3 weeks, and local radiotherapy focusing on anterior mediastinum.

However, the patient’s neurologic symptoms persisted after one course of ADOC treatment. Under the diagnosis of paraneoplastic-related Isaacs’ syndrome, he then received a 5-day course of intravenous immunoglobulin (IVIG, total 2 g/kg). Medications including tizanidine, baclofen, gabapentin, phenytoin, oxycodone, oxcarbazepine, fentanyl, codeine, and morphine were tried for muscle cramp and pain control. Mexiletine was also added for his cramps. However, his symptoms including pain, muscle twitching, and cramps persisted. His muscle cramp and pain improved only after high dose prednisolone treatment (50 mg/day) and then gradually subsided 4 months later.

Follow-up chest CT after four courses of ADOC treatment showed that thymoma at anterior mediastinum regressed partially (8.7 cm → 4.5 cm) and the right pleural masses disappeared totally. Follow-up anti-LGI1 and anti-CASPR2 autoantibodies also turned negative. The high rate RNS test showed no more incremental change. (Table 1).

His steroid dose was tapered in the next 5 months. Some muscle twitching showed up again when the prednisolone was tapered to 5 mg in three out of every seven days. Follow-up serum VGKC complex antibodies assay showed positive LGI1 antibody (1:10) while CASPR2 antibody remained undetectable. Chest CT showed an enlarged anterior mass (4.5 cm → 5.7 cm), either post-radiation changes or disease progression. (Table 1) Prednisolone was increased to 60 mg per day, meanwhile, chemotherapy with ADOC continued. His symptoms then subsided again.

## Discussion and conclusions

Isaacs’ syndrome is one of the PNH syndromes, the others including cramp-fasciculation syndrome and Morvan syndrome. Those patients only with myalgia and cramps without weakness, neuropathy, nor central nervous system (CNS) involvement should be categorized into cramp-fasciculation syndrome, which indicates a benign condition. In addition to cramps and fasciculation, unique findings of EMG including neuromyotonia and myokymia are characteristics of Isaacs’ syndrome. Other symptoms including muscle weakness, dysautonomia, and sensory involvement could also be found in the Isaacs’ syndrome. Furthermore, Isaacs’ syndrome is usually associated with paraneoplastic VGKC-complex autoantibodies. In patients with Morvan syndrome, encephalopathy symptoms like confusion and drowsiness could also be found apart from the peripheral nerve symptoms and dysautonomia [1]. In our case, general muscle stiffness, twitching, cramps, neuropathic pain, myokymia, and weakness were noted, and EMG showed neuromyotonia and myokymia. There were no CNS symptoms including hallucinations, agitation, delirium, amnesia, or confusion. Therefore, Isaacs’ syndrome is the most likely in our patient, though without obvious autonomic dysfunction.

Isaacs’ syndrome is now recognized as an autoimmune or paraneoplastic syndrome [2]. Various associated malignancies have been reported, including thymoma, lung cancer, Hodgkin lymphoma, plasmacytoma, lymphoblastic lymphoma, hemangioblastoma, ovarian cancer, and bladder cancer [5]. In these malignancies, thymoma and small cell lung cancer are tumors most commonly seen in patients with Isaacs’ syndrome [6]. In thymoma patients, Isaacs’ syndrome was the second frequent paraneoplastic neurologic disease (3.5%), secondary to myasthenia gravis (MG) (38%) [7]. Isaacs’ syndrome usually coexisted with other autoimmune disorders mostly MG in thymoma patients [8]. In literature, thymoma patients associated with Isaacs’ syndrome without MG or other paraneoplastic syndromes were rare (Table 2). Hence, our case demonstrated that Isaacs’ syndrome could be the initial and only neuromuscular manifestation of malignant thymoma.

**Table 2:** Review previous case series associated with thymoma and Isaacs’ syndrome

Age	33 [9]	42 [10]	46 [11]	46 [12]	48 [13]	53[14]	65 [15]	65 [16]	68 [10]
Sex	Male	Male	Male	Male	Male	Male	Male	Male	Male
Thymoma	Yes	Yes	Yes	Yes	Yes	Yes	Yes	Yes	Yes
Stage (AJCC^1^)	Stage IV	Stage IV	Stage IV	Stage IV	Stage IV	Stage IV	Stage IIIB	Stage IV	No data
Isaacs’ syndrome	Yes	Yes	Yes	Yes	Yes	Yes	Yes	Yes	Yes
Myasthenia gravis	Yes	Yes	Yes	Yes	Yes	Yes	Yes	Not mentioned	No
Other paraneoplastic syndromes	–	–	Pure red cell aplasia	–	Pure red cell aplasia	–	–	–	–
AchR^2^ antibody	Positive	Positive	Not mentioned	Positive	Positive	Not test	Yes	No test	Yes
VGKC^3^/VGKC-complex antibody	Negative	No test	CASPR2^a^	VGKC	VGKC	LGI1^b^ and CASPR2	No test	No test	No test
Other antibodies	–	–	–	–	Antistriatal antibodies	Antistriatal antibody	–	–	–
Thymoma treatment	Thymectomy Chemotherapy Radiotherapy Cytoreductive surgery, HITOC^4^ with cisplatin	Thymectomy Splenectomy	Thymectomy Chemotherapy Radiotherapy	Thymectomy Chemotherapy Radiotherapy	Thymectomy Chemotherapy Radiotherapy	Thymectomy Thoracotomy Pleurectomy Chemotherapy Photodynamic therapy	Thymectomy	Thymectomy Radiotherapy	Subtotal thymectomy Radiotherapy
Isaacs’ syndrome treatment *Poor response*	Phenytoin Baclofen, Tacrolimus Plasma exchange	–	Prednisolone (40 mg/day), Phenytoin, Baclofen, DFPP^5^	Phenytoin (300 mg/day) Carbamazepine (600 g/day)	–	Gabapentin High-dose methylprednisolone IVIG^6^	IVIG (also for MG crisis) with partial improvement	–	–
*Good response*	Carbamazepine	Periodic plasmapheresis Immunosuppressant	Rituximab (600 mg)	Azathioprine Steroids Plasma exchanges (also for MG crisis)	–	–	Carbamazepine	–	Plasma exchange
Follow-up	Symptoms relieved after thymoma treatment. No symptoms and no tumor recurrence in the next 1.5 years.	The patient died due to metastatic disease a few months later.	Moderate decrease of CASPR2 antibody and free of myokymia two months after initiation of rituximab.	During steroid and cytotoxic therapy, anti-VGKC antibodies fell. VGKC antibodies increased after the withdrawal of cytotoxic treatment. The patient died from cardiopulmonary arrest.	The patient died 5 months later due to acute liver failure.	Symptoms improved after chemotherapy.	Symptoms still existed but were under control by carbamazepine.	No symptoms exist after radiotherapy.	The patient died 8 months after the operation. The autopsy was declined.

*Abbreviations*: *AJCC* American Joint Committee on Cancer, *AchR* acetylcholine receptor, *VGKC* voltage-gated potassium channels, *HITOC* Hyperthermic intrathoracic perfusion chemotherapy, *DFPP* Double-filtration plasmapheresis, *IVIG* intravenous immunoglobulin,

^a^ CAPSPR2: contactin-associated protein-like 2

^b^ *LGI1* leucine-rich glioma-inactivated 1

In the review of Bernard et al, patients with thymoma and concurrent autoimmune disease were divided into “MG” and “autoimmune disease other than MG” groups. They found that patients in the “MG” group were frequently in the early Masaoka stage (I and II), whereas patients in the “autoimmune disease other than MG” group were more frequently in the late-stage (IV) category [7]. Consistent with their findings, our patient who had Isaacs’ syndrome (autoimmune disease other than MG) was found to have late-stage thymoma (including both AJCC and Masaoka stages).

Isaacs’ syndrome is frequently associated with VGKC complex antibodies, which could be found in 38–50% of patients [7]. VGKC are membrane channels that can stabilize the resting potential and decrease the repetitive firing of neurons [17]. Previous data showed that only a few antibodies attack against the potassium channel subunits directly, and most of these antibodies targeted VGKC-complex proteins, like LGI1, CASPR2, and contactin-2 [18]. Our patient was found to have both LGI1 and CASPR2 antibodies. These two antibodies are associated with variable phenotypes. LGI1 antibodies primarily present with CNS symptoms [19], especially autoimmune encephalitis, which could involve both the limbic and extralimbic systems [17]. There are 31% of LGI antibody-positive patients who display peripheral nerve system involvement including hyperexcitability and neuropathic pain [20]. Patients with CASPR2 antibodies could present both CNS and PNS symptoms including neuromyotonia, Morvan syndrome, and limbic or more extensive encephalitis, yet PNS symptoms are more common [19–21] Coexistence of both LGI1 and CASPR2 antibodies in “double positive” individual is not uncommon [4]. In a recent review of double-positive antibodies patients, their clinical presentations could be associated with both antigenic targets but more weights towards to CASPR2 antibody disease spectrum, with PNS involvement being the dominant clinical symptoms. Double-positive antibodies patients are frequently associated with underlying thymoma [4].

In literature, only 2 case reports were found concerning the long-term correlation between clinical symptoms and antibodies titer (Table 2). One case report showed the correlation between Isaacs’ syndrome and anti-VGKC antibodies titer [12]. The other showed CASPR2 antibodies titer was in parallel with clinical symptoms [11]. In our patient, both LGI1 and CASPR2 antibodies were detected when he presented with Isaacs’ syndrome. However, follow-up serum antibody assay showed that his Isaacs’ syndrome correlated only with LGI1 antibody titer. Hence to our acknowledgement, this is the first case report showing the correlation between Isaacs’ syndrome and LGI1 antibodies titer in a thymoma patient with double-positive antibodies. Moreover, we also noted that his thymoma size correlated to LGI1 antibodies titer. This implies that the VGKC complex antibody, LGI1 antibody in our case, probably is a useful tool for long-term follow-up of Isaacs’ syndrome and thymoma condition. The reason why both LGI1 and CASPR2 antibodies could induce the same clinical symptoms of Isaacs’ syndrome remained unclear. It is reasonable to assume that these two autoimmune antibodies might share common intermediate molecules. Binks et al. suggested maybe a common injury or immunological predisposition could cause epitope spread and the preferential presentation of the neighboring VGKC complexed antigens [4].

Isaacs’ syndrome is a paraneoplastic autoimmune disorder likely through VGKC complex autoimmune antibody. Thus, management of such patients aims at two parts: symptomatic relief of Isaacs’ syndrome and treatment of underlying malignancy. Previous reports showed that in patients with antibodies-associated Isaacs’ syndromes, immunotherapies (IVIG, plasma exchange, and steroid) are the mainstay of treatment [4] and various responses were reported (Table 2). Our patient demonstrates a good response of Isaacs’ syndrome to steroid therapy. His painful muscle cramps rapidly improved with a high dose of steroids accompanied by decreasing autoantibody titer. However, his symptoms showed up again when the steroid was tapered, with an elevation of autoantibodies titer again. It is worth noting that, though steroid response could be rapid and dramatic, the risk of symptoms recurrence after rapid reduction of steroid does exist and should be carefully observed in such patients. IVIG therapy was not effective at all for our patient. Second, treatment toward underlying malignancy is crucial. According to the report of Ozcakar et al., Isaacs’ syndrome might improve after thymectomy and radiotherapy for the residual tumor without immunosuppressive therapy [16]. Oncological treatment including chemotherapy and radiotherapy also significantly improves the neurological diseases in patients with thymoma-related PNH syndrome [9, 12, 22]. In summary, we recommend high-dose steroids should be first tried for all patients with Isaacs’ syndrome, and long-term immunosuppressant therapy may be necessary for the maintenance of symptomatic relief. Besides, for patients with non-operable thymoma, aggressive CCRT should be considered.

There is only one case report showing presynaptic neuromuscular dysfunction in patients with thymoma-related Isaacs’ syndrome, based on a 180% increment after 15 s of isometric exercise [10]. That patient had a negative voltage-gated calcium channels (VGCC) antibody, but the VGKC complex antibody was not tested. In our patient, presynaptic neuromuscular dysfunction was based on an abnormal high rate RNS result. However, we did not check the VGCC antibody. Although our patient had mild proximal muscle weakness, typical symptoms for Lambert-Eaton myasthenic syndrome (LEMS) such as autonomic dysfunction and areflexia were lacking. After CCRT and steroid therapy in our patient, no more abnormal incremental response was noted on follow-up high rate RNS test. Although rare, there are a few case reports that described the correlation between thymoma and LEMS. [22–24]. Therefore, we presume that this electrophysiological evidence of presynaptic dysfunction is related to the thymoma itself. Whether VGKC-complex antibodies themselves have a wide regulation on neurotransmitters in acetylcholine transmission, [10] or the coexistence of unknown antibodies other than VGCC and VGKC against neuromuscular junction, or these antibodies share the common downstream pathway, is still unclear.

Isaacs’ syndrome could be the initial and only presentation of malignant thymoma, especially in the late tumor stage. VGKC-complex autoantibody assay including LGI1 and CASPR2 is of clinical importance and longitudinal follow-up of these autoantibodies could provide valuable information. Treatment should aim at two parts, Isaacs’ syndrome, and underlying thymoma. High dose steroids should be tried first, and long-term maintenance dose may be necessary for the treatment of Isaacs’ syndrome. Meanwhile, those with non-operable thymoma demand concurrent CCRT.

## Data Availability

All data related to this case report are documented within this manuscript.

## Abbreviations

ADOC: Doxorubicin, cisplatin, vincristine and cyclophosphamide
AJCC: American Joint Committee on Cancer
CASPR2: Contactin-associated protein 2
CCRT: Concurrent chemoradiotherapy
CNS: Central nervous system
CT: Computed tomography
EMG: Electromyography
IVIG: Intravenous immunoglobulin
LEMS: Lambert-Eaton myasthenic syndrome
LGI1: Leucine-rich glioma-inactivated 1
MG: Myasthenia gravis
PET: Positron emission tomography
PNH: Peripheral nerve hyperexcitability
RNS: Repetitive nerve stimulation
VGCC: Voltage-gated calcium channel
VGKC: Voltage-gated potassium channels
WHO: World Health Organization
